# A Comprehensive Investigation on 3D Printing of Polyamide 11 and Thermoplastic Polyurethane via Multi Jet Fusion

**DOI:** 10.3390/polym13132139

**Published:** 2021-06-29

**Authors:** Wei Shian Tey, Chao Cai, Kun Zhou

**Affiliations:** 1 HP-NTU Digital Manufacturing Corporate Lab, School of Mechanical and Aerospace Engineering, Nanyang Technological University, Singapore 639798, Singapore; weishian001@e.ntu.edu.sg (W.S.T.); chaocai@hust.edu.cn (C.C.); 2 State Key Laboratory of Materials Processing and Die & Mould Technology, Huazhong University of Science and Technology, Wuhan 430074, China; 3 Singapore Centre for 3D Printing, School of Mechanical and Aerospace Engineering, Nanyang Technological University, Singapore 639798, Singapore

**Keywords:** powder bed fusion, Multi Jet Fusion, Polyamide 11, thermoplastic polyurethane, mechanical properties

## Abstract

Multi Jet Fusion (MJF) is a recently developed polymeric powder bed fusion (PBF) additive manufacturing technique that has received considerable attention in the industrial and scientific community due to its ability to fabricate functional and complex polymeric parts efficiently. In this work, a systematic characterization of the physicochemical properties of MJF-certified polyamide 11 (PA11) and thermoplastic polyurethane (TPU) powder was conducted. The mechanical performance and print quality of the specimens printed using both powders were then evaluated. Both PA11 and TPU powders showed irregular morphology with sharp features and had broad particle size distribution, but such features did not impair their printability significantly. According to the DSC scans, the PA11 specimen exhibited two endothermic peaks, while the TPU specimen exhibited a broad endothermic peak (116–150 °C). The PA11 specimens possessed the highest tensile strength in the *Z* orientation, as opposed to the TPU specimens which possessed the lowest tensile strength along the same orientation. The flexural properties of the PA11 and TPU specimens displayed a similar anisotropy where the flexural strength was highest in the *Z* orientation and lowest in the *X* orientation. The porosity values of both the PA11 and the TPU specimens were observed to be the lowest in the *Z* orientation and highest in the *X* orientation, which was the opposite of the trend observed for the flexural strength of the specimens. The PA11 specimen possessed a low coefficient of friction (COF) of 0.13 and wear rate of 8.68 × 10^−5^ mm^3^/Nm as compared to the TPU specimen, which had a COF of 0.55 and wear rate of 0.012 mm^3^/Nm. The PA11 specimens generally had lower roughness values on their surfaces (*R*_a_ < 25 μm), while the TPU specimens had much rougher surfaces (*R*_a_ > 40 μm). This investigation aims to uncover and explain phenomena that are unique to the MJF process of PA11 and TPU while also serving as a benchmark against similar polymeric parts printed using other PBF processes.

## 1. Introduction

Additive manufacturing (AM) is a family of near-net-shape manufacturing processes that creates objects using computer-aided designs through a layer-by-layer accumulation of material feedstock [1]. The powder bed fusion (PBF) process, which is classified as one of the seven main categories of AM by ASTM, has garnered substantial interest from industries and the scientific community due to its ability to fabricate geometrically complex parts using any fusible powder-form materials theoretically without any need for support structures [2,3].

Multi Jet Fusion (MJF) is a recently developed polymeric PBF process patented by HP Inc. in 2014 and commercialized in 2016. During the MJF process, functional polymeric parts can be produced by heating and fusing selected areas in the polymer powder bed through the combination of ink agents and infrared (IR) lamps. Two different ink agents, i.e., fusing and detailing agents, are jetted into the designated areas in the powder bed through thermal inkjet printheads with a voxel resolution. The proprietary fusing agent consists of black radiation-absorbing material and can enhance the optical-to-thermal energy conversion to melt the powder particles where the fusing agent is jetted on. The detailing agent, which mainly comprises of a non-radiation absorbing water-based material, is simultaneously jetted around the boundaries of parts to decrease the local temperature of the contours to avoid the fusion of powder particles surrounding the contours. The powder bed is then heated by moving overhead IR lamps across the powder bed, and the powder bed regions jetted with the fusing agent will be fused to form object parts, while the fusion of powder surrounding the boundary of parts is inhibited by the detailing agent. The combination of these two ink agents allows the fabrication of dense functional parts with superior edge definition and surface finish. The utilization of a planar fusion mode contributes to the printing speed of MJF being significantly faster than other polymeric PBF processes, such as selective laser sintering (SLS), which adopts a point-by-point laser scanning mode. Xu et al. [4] reported that these differences in processing principles resulted in the printing speed of MJF to be almost ten times faster than that of SLS.

Polyamide 12 (PA12) has been the most commonly used polymer powder in polymeric PBF processes such as MJF and SLS [5,6]. A systematic benchmark study on the comparison between SLS and MJF manufacturing techniques of PA12 has been revealed in our previous publication [7]. Besides PA12, HP Inc. has also developed and commercialized two other promising polymer powders, HP 3D HR PA11 and BASF Ultrasint TPU01, to broaden the industrial applications of MJF.

Polyamide 11 (PA11) is a 100% bio-based polyamide synthesized from a renewable source (castor oil) and has a lower carbon footprint compared to the petrol-based PA12 [8,9]. It also possesses a good strength-to-weight ratio, high fatigue resistance, and excellent chemical and aging resistance [10,11,12]. These advantages make it the second most widely used polymer PBF material in aerospace, automotive, pneumatic, and electrical applications [13]. For instance, PA11 has been utilized in the additive manufacturing of more than 2000 environmental control system air ducts by Boeing for their F/A-18 Super Hornet program. The printed components significantly reduced the weight and cost of the rotomolded system because the manufacturing flexibility of this technology allowed engineers to design the components with an integral structure and optimized topology [14].

Thermoplastic polyurethane (TPU) is an elastomer with a good combination of mechanical strength and flexibility. TPU consists of hard segments, which are formed by adding a chain extender to isocyanate, and soft segments, which are made up of either polyether or polyester chains. The hard segments contribute to the strength of TPU by functioning as both physical crosslinks and reinforcement within the elastomer, while the soft segments contribute to the flexibility and elongation [15]. By changing the phase composition and heating conditions, the mechanical properties of TPU can be altered. This unique property of TPU makes it an ideal candidate for non-commodity markets such as custom-made footwear and sporting goods [16]. For example, TPU was used to fabricate the flexible honeycomb midsoles in the Zante Generate running shoes by New Balance using 3D printing [17].

There have been several studies conducted on the characterization of MJF-printed parts that uncovered specific properties that are unique to the MJF process. For example, it was reported in several publications that MJF-printed PA12 specimens printed along the vertical direction possessed higher tensile and flexural properties as opposed to specimens printed horizontally [6,7,18,19]. It was also observed in several DSC scans that the MJF-printed PA12 specimens did not exhibit bimodal endothermic peaks, unlike in SLS specimens where bimodal endothermic peaks were commonly observed [7,19,20,21]. However, these studies were all conducted on PA12 and works on other commercially available MJF materials are scarce. It is therefore essential to investigate other commercially available MJF materials to determine if these unique properties are consistent among all MJF materials, as well as to gain a deeper understanding of the underlying mechanisms that create these unique properties in MJF parts.

To date, there are only a few works published on the characterization of commercially available MJF materials other than PA12. Lee et al. [22] investigated the effect of build orientation on the part porosity and mechanical properties of MJF-printed PA11. Pandelidi et al. [23] studied the effect of powder refresh ratios on the thermal and mechanical properties of MJF-printed PA11 parts. Šafka et al. [24] conducted a comprehensive characterization on the mechanical properties of MJF-printed polypropylene printed along seven major print orientations. To the authors’ knowledge, there are no publications on MJF-printed TPU parts to date.

While there are publications on the characterization of MJF-printed PA11, the published reports do not provide a full evaluation of the physical, chemical, thermal, mechanical, and surface properties of the powder and printed parts. Therefore, this work attempts to provide a comprehensive characterization on the physicochemical properties of both MJF-certified PA11 and TPU powders, as well as to evaluate the mechanical properties and surface quality of their respective printed parts. The purpose of this study is to unveil the underlying mechanisms behind the phenomena unique to the MJF process of PA11 and TPU parts, as well as to compare the mechanical properties of the printed parts against similar parts printed using other PBF processes.

## 2. Material and Methods

### 2.1. Powder Materials and Printing Equipment

HP 3D High Reusability PA11 (defined as PA11 hereafter) powder was employed for the printing of PA11 specimens using an MJF 4200 printer, and BASF Ultrasint TPU01 (defined as TPU hereafter) powder was adopted to print TPU specimens using an MJF 5200 printer. All the specimens were printed in the ‘Balanced’ print mode. The PA11 specimens were printed with a virgin/used powder mix ratio of 30:70, while the TPU specimens were printed with a virgin/used powder mix ratio of 20:80.

**Figure 1:**
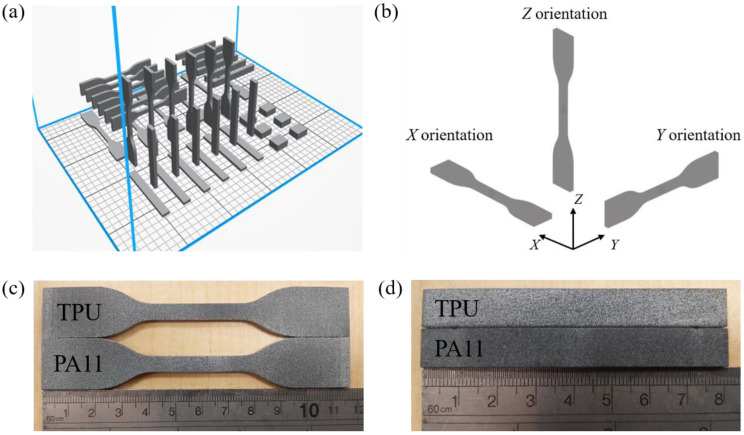
Design and fabrication of MJF-printed specimens: (**a**) print layout, (**b**) build orientation axes in accordance with ISO/ASTM 52921, (**c**) tensile bars and (**d**) flexural bars.

The specimen arrangement in the printing chamber is illustrated in Figure 1a. Tensile and flexural specimens were printed in accordance with the ASTM D638-10 and ISO178 standards, respectively. Six tensile and flexural specimens were printed in *X*, *Y*, and *Z* build orientations as defined by the ISO/ASTM 52921 standard (Figure 1b) to investigate the mechanical anisotropy of the printed specimens. Five rectangular specimens with a designed dimension of 20 × 16 × 10 mm^3^ were also printed for the surface roughness evaluation of their top, front, and side surfaces. All the printed specimens were bead blasted (Kompac 750, Abrasive Engineering, Singapore) with glass beads to remove adherent powder particles.

### 2.2. Powder and Specimen Characterization

The particle size distribution of the PA11 and TPU powders were evaluated using a laser diffraction particle analyzer (Mastersizer 2000, Malvern Panalytical, Malvern, UK). The morphology of the PA11 and TPU powders was observed using a JSM-5600 scanning electron microscope (SEM) at an acceleration voltage of 10 kV. Differential scanning calorimetry (DSC) tests were conducted on the PA11 and TPU powders and specimens using a DSC-Q200 differential scanning calorimeter (TA Instruments, New Castle, DE, America) to evaluate their thermal properties. DSC specimens of weight 10 ± 1 mg were tested under an increasing temperature ramp from 25 °C to 250 °C at a rate of 10 °C/min followed by cooling from 250 to 25 °C at a rate of 10 °C/min. DSC measurements were carried out with a nitrogen flow rate of 40 mL/min and repeated 3 times for data reliability. The melting enthalpy of the specimens was calculated by integrating the endothermic peak using the TA instruments TRIOS software, and the crystallinity *X*_m_ of the PA11 specimens can be calculated by:

(1) $$X_{m}=\frac{∆H_{m}}{∆H_{m}^{0}}\times 100\%$$

where ∆$H_{m}$ is the melting enthalpy calculated from the endothermic peak (J/g), and $∆H_{m}^{0}$ is the theoretical enthalpy for a 100% crystalline PA11 matrix ($∆H_{m}^{0}=$ 226.4 J/g) [25,26]. The theoretical enthalpy for 100% crystalline TPU matrix is unknown as the phase components and composition of the TPU powder are kept confidential. Hence, the crystallinity of the TPU powder and the TPU specimen cannot be calculated.

X-ray diffraction (XRD) measurements (XRD-6000, Shimadzu, Kyoto, Japan) were conducted on both the powders and the specimens to identify their phase constitutions using Cu Kα radiation in a 2θ range of 5–35° at a scan speed of 2°/min. The Fourier-transform infrared spectra of the PA11 and TPU powders were analyzed using the IR Prestige 21 (Shimadzu, Kyoto, Japan) for functional group identification. X-ray photoelectron spectroscopy (XPS) scans were performed using a Kratos AXIS Supra, Manchester, UK, to identify the surface chemical states of both powders and specimens. An initial wide scan was conducted to determine the surface chemical composition of the specimens, and high-resolution scans were taken afterwards to observe the composition of chemical bond types at specific regions of interest.

The porosity of the specimens printed in the *X*, *Y,* and *Z* build orientation was evaluated using a Bruker Skyscan 1173 micro-computed tomography (micro-CT) machine. A cuboid of dimension 10 × 4 × 4 mm^3^ was imaged from the middle portion of the PA11 and TPU flexural specimens. A source voltage of 80 kV, source current of 100 μA, rotation step of 0.2°, exposure of 1200 ms and pixel size of 7.1 μm were used for the imaging of all the specimens. The images were then reconstructed and analyzed using the CTAn software. To ensure a fair comparison, similar thresholding values were utilized for the analysis (lower = 65, upper = 255) of all specimens.

### 2.3. Mechanical Performance and Surface Topography

The mechanical performance of the PA11 and TPU specimens was assessed through tensile, flexural, and tribological tests. The tensile testing was performed using a Shimadzu AGX 10 kN universal tester at a strain speed of 10 mm/min. The fracture surfaces of the tensile bars were then observed using SEM. The flexural tests were performed on a universal tester (Instron 5569 50 kN, Instron, Norwood, MA, USA) at a testing speed of 2 mm/min to a maximum deflection of 20 mm, with the distance between the two supports being 64 mm. Linear reciprocating wear tests were conducted on the top surface of both the PA11 and TPU specimens using a UMT-Tribolab wear tester. Specimens were grinded and polished to achieve a flatter surface prior to the wear test. Each specimen was then secured in the sample holder, and a Si_3_N_4_ ball of radius 3.175 mm was loaded against the specimen at a normal load of 35 N. The ball moved in a reciprocating linear motion at a stroke length of 10 mm and a speed of 10 mm/s for 45 min. Thereafter, the wear track of each specimen was observed using a laser confocal microscope (VK X200K, KEYENCE, Osaka, Japan) to determine their wear volume and wear rate. From the wear morphology, the wear scar width was determined, and the wear volume *V* was calculated by:

(2) V=l[(r2sin−1(w2r)−w2(r2−w24)12]+π3[2r3−2r2(r2−w24)12−w24(r2−w24)12]

where *w* is the width of the wear scar (mm), *l* is the stroke length (mm), and *r* is the radius of the ball (mm). The wear rate *k* was then calculated using the following:

(3) $$k=\frac{V}{NL}$$

where *V* is the wear volume (mm^3^), *N* is the normal load applied (*N*), and *L* is the total distance travelled by the pin (m).

The surface morphology and topography of the PA11 and TPU specimens were measured using the laser confocal scanning microscope (VK X200, KEYENCE, Osaka, Japan). Laser and optical scans were performed on a 4.3 × 3.2 mm^2^ area in the middle portion of each specimen surface to measure the surface roughness value *R*_a_. The surface roughness calculations of each surface were repeated and averaged across 4 specimens to ensure data reliability.

## 3. Results and Discussion

### 3.1. Material Feedstock Characterization

**Figure 2:**
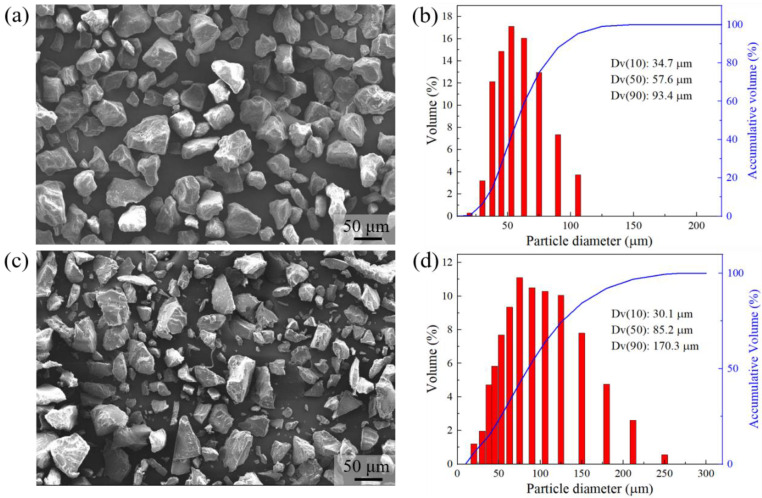
Granulometric morphology and powder size distribution of powders: (**a**,**b**) PA11 and (**c**,**d**) TPU powders.

The granulometric distribution and morphology of both PA11 and TPU powders are shown in Figure 2. Both powders consisted of irregularly shaped particles with sharp edges, which suggested that both powders were prepared using cryogenic milling [27]. The observed powder morphology of both the PA11 and TPU powders were in line with publications [16,28], where the PA11 and TPU powders were cryogenically milled. Compared to the PA11 and TPU powders, the HP 3D HR PA12 powder presented fascinating near-spherical particles with smooth edges, which suggested that it was produced using the dissolution-precipitation method, a powder preparation process that produces powder particles of similar morphology [28]. The TPU powder cannot be prepared through the dissolution-precipitation method because there is currently no suitable solvent that can dissolve the TPU particles efficiently. While the PA11 powder can be prepared through dissolution-precipitation, this process is less cost-effective as compared to cryogenic milling. The PA11 powder had particle sizes ranging from 30–110 μm with a D_v_(50) of 57.6 μm, while the TPU powder showed a broader particle size range of 20–250 μm with a D_v_(50) of 85.2 μm.

### 3.2. Thermal and Chemical Analysis

**Figure 3:**
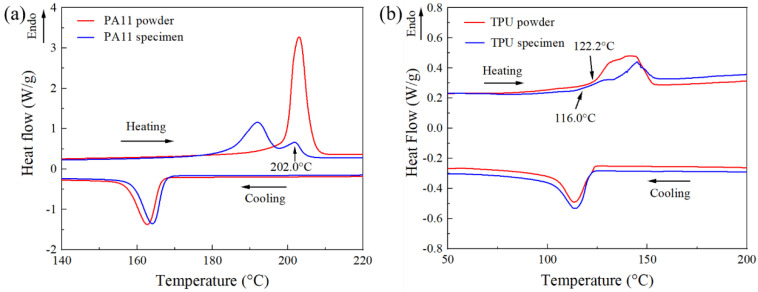
DSC curves of powders and specimens: (**a**) PA11 and (**b**) TPU.

From the DSC curves, various thermal properties such as the onset melting temperature *T*_om_, the peak melting temperature *T*_pm_, the onset crystallization temperature *T*_oc_, and the peak crystallization temperature *T*_pc_ were extracted and summarized in Table 1. The DSC curve of the PA11 powder (Figure 3a) presented a distinct endothermic peak and exothermic peak at 202.9 °C and 162.7 °C, respectively. The large difference between *T*_om_ and *T*_oc_ signified a large supercooling region, which is a desirable property for material feedstock in PBF processes as it can significantly reduce crystallization of the polymer melt during the fabrication process. The reduced crystallization of printed polymeric parts during the printing process helped to decrease the shrinkage of the printed parts, hence relieving the accumulation of internal stresses within the parts and guaranteeing the processability of the polymeric material.

The DSC curve of the PA11 specimen showed two endothermic peaks, a main endothermic peak at 192.0 °C, and a smaller shoulder endothermic peak at 201.8 °C. The presence of a shoulder peak is a common phenomenon in SLS-printed PA12, but not in MJF-printed PA12 [7,20,21]. It has been confirmed that the shoulder peak was a result of the partial un-melting of powder particles. This indicated that a portion of the PA11 powder particles, particularly the larger particles, could not melt completely due to insufficient heat energy received during the MJF fusing process, which resulted in the presence of un-melted powder particle cores in the polymer parts [29]. This conclusion was further affirmed by the similarities between the shoulder peak (201.8 °C) and the *T*_pm_ of the powder (202.9 °C). The crystallinity of the PA11 specimen was calculated to be 26.2%, which was higher than that of the SLS-printed PA11 (19.8%) [30]. The increased crystallinity in the MJF-printed PA11 specimens could stem from the carbon black that was deposited into the specimens through the fusing agent. These carbon blacks acted as nucleation sites, thereby accelerating the crystallization process.

The DSC curves of the TPU powder and the TPU specimen (Figure 3b) exhibited broad endothermic peaks, which are commonly observed in TPU materials because of their complex morphology [15]. The *T*_om_ of the TPU powder was observed to be higher than that of the TPU specimen. The lower *T*_om_ of the TPU specimen could be caused by the disordering of the hard segment crystallites with short-range order that were formed during the cooling and annealing process [15]. The *T*_om_ (122.2 °C) of the TPU powder was close to its *T*_oc_ (123.9 °C), which could be detrimental to the printing process as the preheating temperature should be higher than the *T*_oc_ to prevent any part warpage from residual stresses. However, there was no part warpage or shrinkage observed in the TPU specimens. This observation was also shared by Verbelen et al. [16] who observed no visible warpage in TPU samples printed using SLS. This could be due to the minimal shrinkage experienced by the hard segments during recrystallization, which in turn caused minimal part distortion. This implied that the absence of a sintering window will not have a significantly adverse effect on the TPU processability for the MJF process as the recrystallization of TPU parts during the printing process does not warp the printed parts severely.

**Figure 4:**
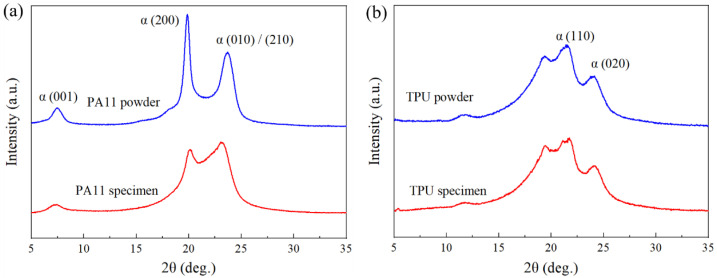
XRD analyses of powder and specimen: (**a**) PA11 and (**b**) TPU.

The XRD patterns of the PA11 powder and PA11 specimen are shown in Figure 4a. Both diffraction patterns reflected similar diffraction peaks at 7.4°, 20.0°, and 23.3°, which corresponded to the triclinic α-form (001), (200), and (010)/(210) planes, respectively [30,31]. However, the diffraction pattern of the PA11 specimen showed a smaller gap and a less prominent separation between the main diffraction peaks as compared to the PA11 powder. This diffraction pattern corresponded to the triclinic α’ phase, which was formed upon cooling the polymer melt (pseudohexagonal δ phase) below the Brill transition temperature after the MJF process [32]. In contrast to the α phase, which remains stable up to its melting temperature, the α’ crystals will transform into δ crystals above the Brill transition temperature. However, as the melting temperature of the α’ crystals are lower than that of the δ crystals, a portion of the α’ crystals melt before it can transform to δ phase [31]. This phenomenon could be seen in the DSC curve of PA11 (Figure 3a), where the *T*_om_ of the PA11 specimen was much lower than that of the PA11 powder. The diffraction patterns of both the TPU powder and TPU specimen are shown in Figure 4b. Both the TPU powder and TPU specimen possessed characteristic peaks at 2θ angles of 19.5°, 21.0°, and 24.0°, indicating that no chemical reactions occurred during the printing process. The diffraction peaks at 21.0° and 24.0° were assigned to the (110) and (020) planes of the monoclinic α-form of poly(ethylene adipate) [33].

**Figure 5:**
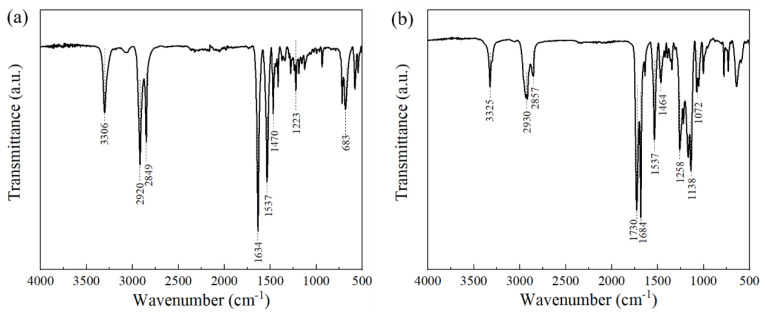
FTIR spectra of powders: (**a**) PA11 and (**b**) TPU.

The infrared spectra of both powders and their respective peak assignments are summarized in Figure 5 and Table 2. The infrared spectra of the PA11 powder exhibited the typical characteristic peaks of PA11 and were similar to the PA11 powders manufactured by Rilsan [25,34]. The infrared spectra of the TPU powder displayed peaks that matched the characteristic absorption bands of polyurethane corresponding to N–H (3325 cm^−1^), C–H (2800–3000 cm^−1^ and 1464 cm^−1^), and the vibration of amide groups (1464 cm^−1^) [35]. The peaks at 1138 cm^−1^ and 1258 cm^−1^ corresponding to the polyether components were also reflected in the spectra of the TPU powder.

**Figure 6:**
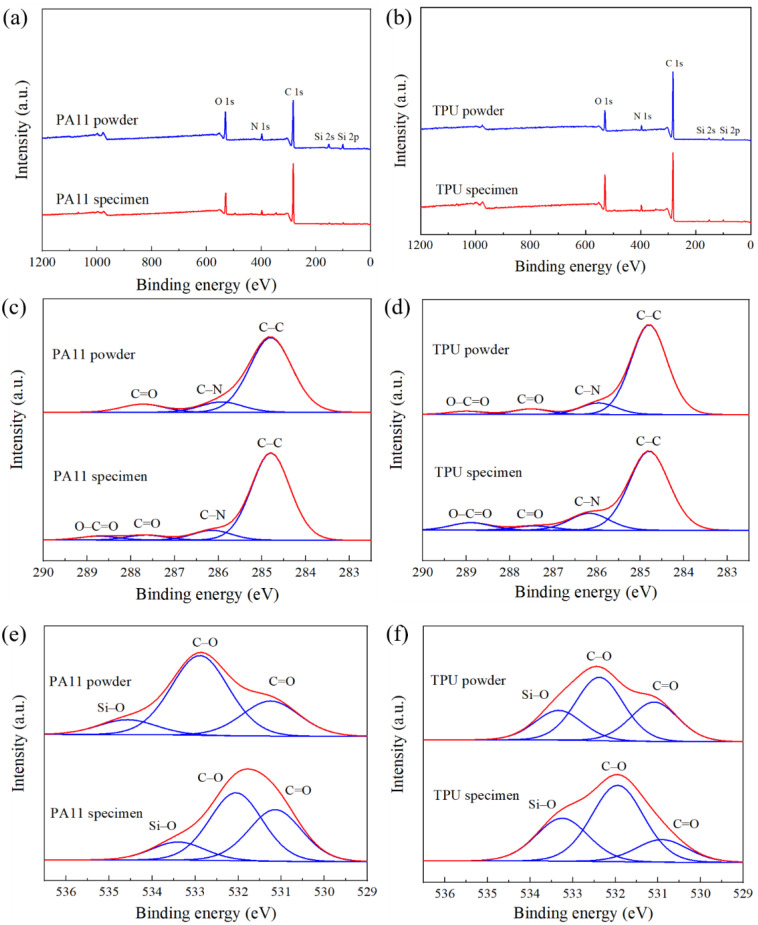
XPS analyses of powders and specimens: (**a**,**b**) wide scan; (**c**,**d**) high-resolution spectra of carbon chemical state; (**e**,**f**) high-resolution spectra of oxygen chemical state.

The XPS spectra of both PA11 and TPU powders and their respective specimens are illustrated in Figure 6a,b respectively. The spectra of both materials reflected three distinct peaks at 530 eV, 398 eV, and 283 eV, which corresponded to the S-shell orbital chemical shift of oxygen, nitrogen, and carbon, respectively. Several minor peaks that were assigned to Si chemical bonds were also observed. The presence of these elements stemmed from the inorganic powdered additives that were added to improve the powder flowability and oxidation resistance of the PA11 and TPU powders [36]. The chemical state of carbon and oxygen in the PA11 and TPU powders and their respective specimens were identified using a high-resolution scan (Figure 6c–f). The composition of the chemical bonds for the PA11 and TPU powders and specimens are summarized in Table 3.

### 3.3. Porosity Evaluation

**Figure 7:**
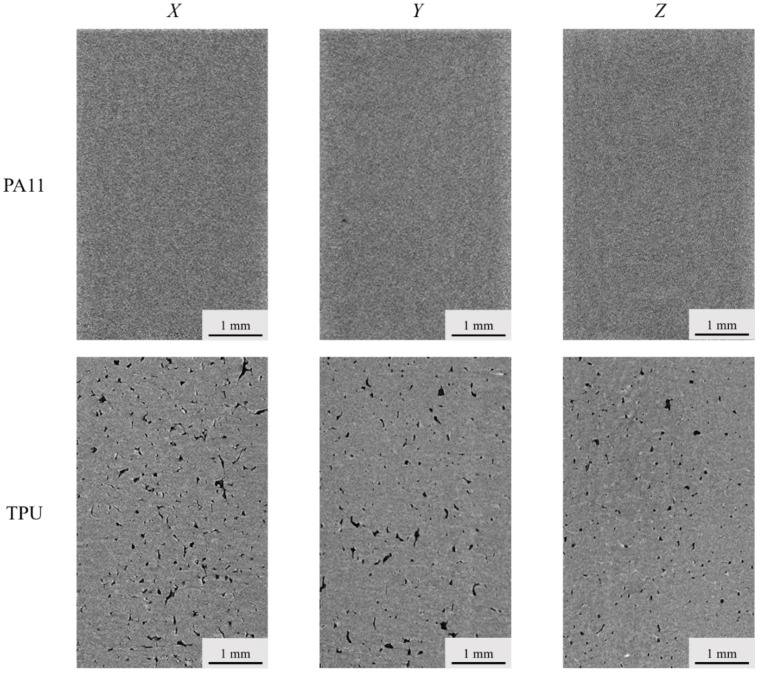
2D images of PA11 and TPU flexural specimens from micro-CT scans.

Micro-CT scans were conducted on the PA11 and TPU flexural specimens of each build orientation to determine the effect of build orientation on part porosity. The respective porosity content of the specimens is summarized in Table 4. Figure 7 illustrates the 2D images acquired from the CT scans of the flexural specimens printed along the *X, Y*, and *Z* orientation for the PA11 and TPU specimens. The porosity of the PA11 specimens (~1%) was relatively lower than that of the TPU specimens, which ranged from 2.45 to 5.45%. The PA11 specimens mainly consisted of very fine micro-sized pores, while much larger irregular pores were observed in the TPU specimens. The porosity content of both the PA11 and TPU specimens observed a similar trend whereby the specimen porosity was lowest in the *Z* orientation and highest in the *X* orientation.

### 3.4. Mechanical Performance

**Figure 8:**
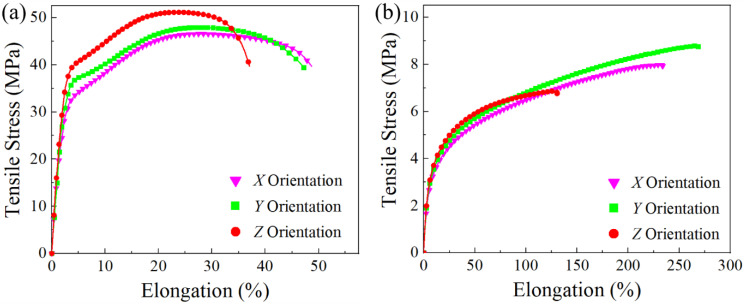
Stress–strain curves of tensile specimens: (**a**) PA11 and (**b**) TPU.

**Figure 9:**
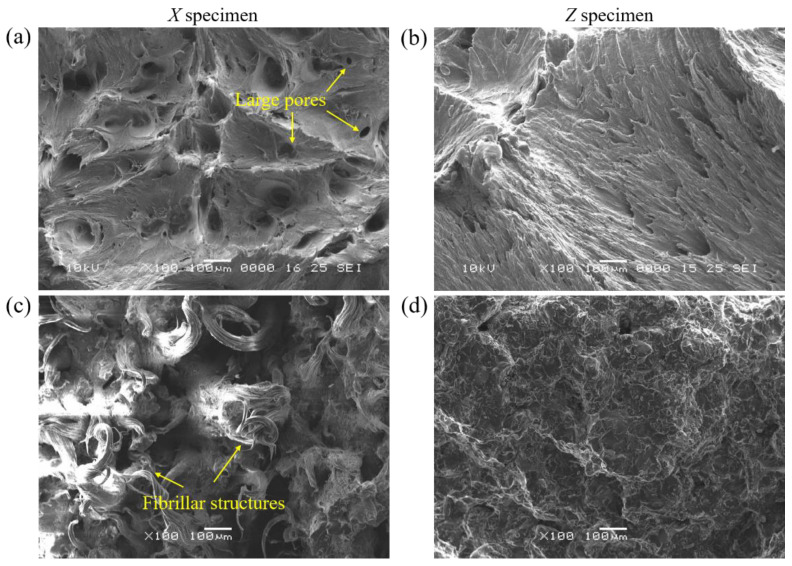
Fracture morphologies of specimens: (**a**,**b**) PA11 and (**c**,**d**) TPU.

The tensile and flexural tests were conducted on the PA11 and TPU specimens in the different build orientations to assess their mechanical performance and anisotropy. Figure 8 shows a tensile property comparison of the PA11 and TPU specimens with respect to the build orientations. The specimens printed along the *X, Y,* and *Z* build orientation are defined as the *X*, *Y,* and *Z* specimens hereafter. Table 5 summarizes the tensile properties of the PA11 and TPU specimens. Obvious anisotropy in the tensile strength of the PA11 specimens was observed. The *X* and *Y* specimens displayed almost identical ultimate tensile strength (UTS) and elongation at break (*ε*_ab_). The *Z* specimens exhibited a significant increase in UTS (50.9 MPa) and elastic modulus (1319.8 MPa), but with a decrease in *ε*_ab_ (32.1%). These results were consistent with several publications [18,19,22], and the enhanced tensile strength of the *Z* specimens could be attributed to the increased material density caused by the weight of the fused powder compressing on the preceding layers in the vertical direction. Moreover, the *Z* specimens also received a higher IR exposure from the increased number of the overhead IR lamp sweeps due to the increased number of layers sliced for the *Z* specimens [22]. The higher IR exposure, coupled with the continuous heat conduction from the carbon black in the fusing agent, enabled a deeper depth of energy penetration that led to the re-melting of the previous layers, hence achieving better interfacial bonding [7]. The observation of fracture surfaces was in line with these analyses. Numerous large pores were present on the fracture surface of the *X* specimen but were not observed in the *Z* specimen fracture surface (Figure 9a,b).

Noticeable anisotropy was observed for the TPU specimens, where the *Z* specimens exhibited the lowest UTS and *ε*_ab_. This phenomenon was the opposite of other MJF-printed materials such as PA11 and PA12, which exhibited the highest UTS in the *Z* specimens. Fibrillar structures were observed on the fracture surface of the *X* specimen (Figure 9c,d), which signified the occurrence of strain hardening that was formed through the stretching and alignment of soft and hard segments parallel to the strain direction. In contrast, the absence of fibrillar structures on the fracture surface of the *Z* specimen indicated that there was limited strain hardening, which could be further confirmed from the tensile curve. The reduction in elongation and strain hardening resulted in a lower tensile strength for the *Z* specimens. As the preheating temperature of the printing process (106 °C) was lower than the *T*_oc_ of the TPU powder, the molten TPU began to crystallize before the end of the printing process. This resulted in poorer mixing between layers and weaker interlayer bonding, thereby causing a reduction in the *ε*_ab_ and limiting the strain hardening of the *Z* specimens.

**Figure 10:**
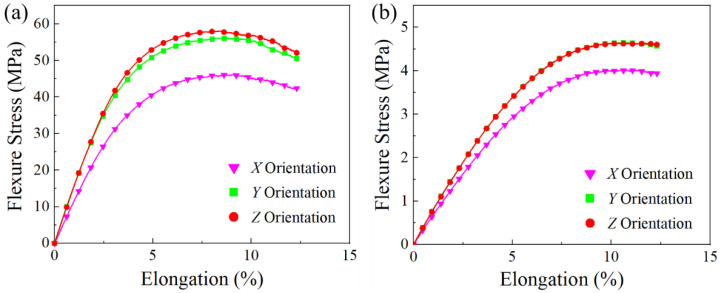
Stress–strain curves of flexural specimens: (**a**) PA11 and (**b**) TPU.

Table 6 summarizes the flexural properties of the PA11 and TPU specimens. Figure 10 shows the flexural property comparison of the PA11 and TPU specimens with respect to build orientation. The flexural performance of the PA11 and TPU specimens followed a similar trend where the flexural strength and modulus of the specimens was highest in the *Z* orientation and lowest in the *X* orientation. This trend was also in line with published reports on MJF-printed PA12 specimens [6,37]. Significant variance was observed in the flexural properties of the specimens printed along the different build orientations, where the *X* specimens exhibited considerably poorer flexural properties as compared to the *Y* and *Z* specimens.

The significant variance in flexural properties could be the result of the varying porosity of specimens printed along different build orientations. The trend observed for the increase in flexural strength was the opposite of the trend observed for the increase in porosity, which implied that the drop in flexural strength was affected by higher part porosity. This result was in line with the work of O’Connor and Dowling [37], who reported that the flexural strength of MJF-printed PA12 and glass bead reinforced PA12 specimens decreased with the increase in their porosity, signifying that the porosity of the parts had a larger impact on the flexural properties of the printed specimens as opposed to its tensile properties.

**Figure 11:**
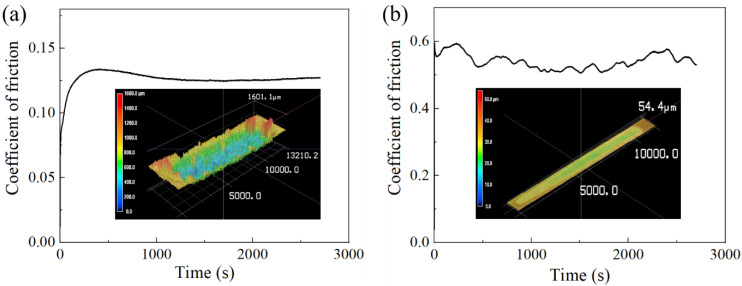
Coefficient of friction and wear morphology of specimens: (**a**) PA11 and (**b**) TPU.

The coefficient of friction (COF) and wear profile of the PA11 and TPU specimens are shown in Figure 11, and the wear volume and wear rate of the PA11 and TPU specimens are compiled in Table 7. The PA11 specimen possessed an average COF of 0.125 and experienced little material loss with a wear rate of 8.68 × 10^−5^ mm^3^/Nm, which indicated that it was highly resistant to sliding abrasion. Initially, the COF of the PA11 specimen was observed to gradually increase until it reached a maximum value before decreasing into a steady value. This transient state observed in the initial stage was characteristic of the running-in period, where the asperities and irregularities of both mating surfaces were removed under the moving load [38]. The PA11 molecules removed under the sliding load were transferred onto the Si_3_N_4_ ball, forming a smooth and thin low-shear transfer film. This film reduced the friction between the mating surfaces, thereby resulting in a subsequent drop in COF to a steady-state value [39].

In contrast, the TPU specimen had an average COF of 0.55 and experienced significant material loss with a wear rate of 0.012 mm^3^/Nm. The high wear rate of TPU is a common trait that is shared among elastomers due to the presence of high adhesive forces between contacts in addition to abrasive forces from asperities. The running-in period of the TPU specimen was significantly shorter compared to that of the PA11 specimen. As TPU has a much higher wear rate than PA11, the irregularities on the TPU surface were removed very quickly under the same load of 35 N, thereby shortening the running-in stage. There was also significant fluctuation in the COF of the TPU specimen, which stemmed from the stick-slip phenomenon that occurred as the sliding direction of the Si_3_N_4_ ball changed [35].

### 3.5. Surface Topology

**Figure 12:**
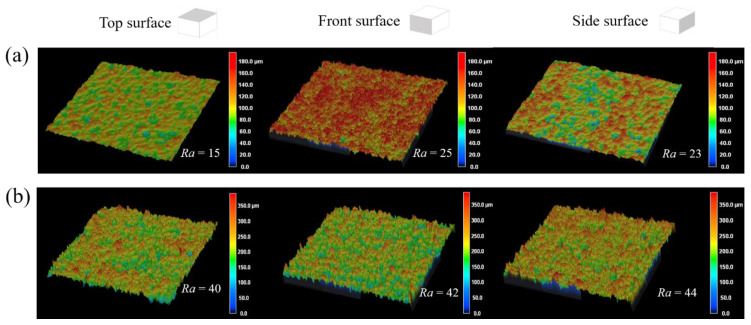
Surface roughness profiles of specimens: (**a**) PA11 and (**b**) TPU.

The surface roughness values and the surface profiles for the top, front, and side surfaces of the PA11 and TPU specimens are shown in Figure 12. The front and side surfaces of the PA11 specimen were notably rougher than the top surface of the specimen. The rougher front and side surfaces originated from the adhesion of surrounding powder to the specimen. The surface roughness on all surfaces of the PA11 specimens was lower than MJF-printed PA12 specimens [7].

The *R*_a_ value on each surface of the TPU specimen appeared to be relatively similar, with no part curvature or distortion observed. However, the surface roughness of each surface of the TPU specimen was high with an average *R*_a_ value of 42. The preheating temperature set for the printing process (106 °C) was nearing the onset melting temperature of the TPU powder. The passing of the fusing lamps, combined with the heat diffusion from the molten part, could cause the portion of the surrounding powder particles to be sintered onto the specimen during the print.

## 4. Conclusions

The results obtained from this characterization study provided insight on the underlying mechanisms behind certain phenomena unique to the MJF process and can be used as a benchmark for comparisons with similar materials fabricated using other processes. Moreover, this study identified the key process parameters that can be further optimized to improve the mechanical performance of existing parts. From this study, the following conclusions can be drawn.

Both PA11 and TPU powders showed irregular morphology with sharp features and had broad particle size distribution. XPS scans indicated the addition of inorganic additives used to improve the flowability of both powders. Unlike the MJF-printed PA12 parts, the DSC curve of the PA11 specimen exhibited two endothermic peaks, which was the result of larger un-melted powder particle cores that remained in the part due to insufficient heating. The DSC curve of the TPU specimen showed a broad endothermic peak, which was characteristic of elastomeric materials due to their complex phase morphology.

There was a strong correlation between the part porosity and the mechanical properties of the specimens (with higher mechanical performance observed at lower part porosity). However, the TPU *Z* specimens possessed the lowest tensile strength despite having the lowest porosity. This was attributed to the limited strain hardening of the material before failure, as observed from the fracture surface.

The PA11 specimen possessed excellent wear resistance with an average COF of 0.125 and a wear rate of 8.68 × 10^−5^ mm^3^/Nm. The TPU specimen had a much higher COF of 0.55 and wear rate of 0.012 mm^3^/Nm due to the large adhesive forces between the contact bodies. The PA11 specimen had front and side surfaces that were much rougher than its top surface, which was caused by the adherence of surrounding powder particles onto the sides of the specimen. The TPU specimen had high average surface roughness for all surfaces, which was the result of the process preheating temperature (106 °C) being too close to the *T*_om_ of the TPU powder.

## Figures and Tables

**Table 1:** Thermal properties of PA11 and TPU specimens.

	*T*_om_ (°C)	*T*_pm_ (°C)	*T*_oc_ (°C)	*T*_pc_ (°C)	Δ*H*_m_ (W/g)	*X*_m_ (%)
PA11 powder	189.2	202.9	168.3	162.7	88.3	39.0
PA11 specimen	178.7	192.0	169.6	163.9	59.3	26.2
TPU powder	122.2	144.4	123.9	113.2	23.9	-
TPU specimen	116.0	144.2	123.6	113.6	11.9	-

**Table 2:** Functional group assignments for both powders.

Material	FT-IR Peak (cm^−1^)	Assignment
PA11	3306	N–H stretching
	2920	CH_2_ asymmetric stretching
	2849	CH_2_ symmetric stretching
	1634	C=O stretching (amide I)
	1537	C–N stretching and C=O in plane bending
	1470	C=O and N-vicinal CH_2_ bending
	1223	C–N stretching
	683	CONH out-of-plane deformation (amide V)
TPU	3325	N–H stretching
	2930	CH_2_ asymmetric stretching
	2857	CH_2_ symmetric stretching
	1730	C=O stretching (amide I)
	1684	C=O stretching (secondary amide)
	1537	N–H bending (amide II)
	1464	C=O and N-vicinal CH_2_ bending
	1258	C–O stretching (alkyl aryl ether)
	1138	C–O stretching (aliphatic ether)
	1072	C–N stretching

**Table 3:** Chemical composition of chemical bonds for both powders and specimens.

	C1s (%)				O1s (%)		
	C–C	C–N	C=O	O–C=O	C=O	C–O	Si–O
PA11 powder	80.1	11.1	8.8	-	27.0	61.5	11.5
PA11 specimen	82.4	9.2	4.7	3.7	37.5	49.3	13.2
TPU powder	81.5	10.5	5.0	3.0	29.7	47.9	22.4
TPU specimen	73.4	15.7	3.9	7.0	15.9	53.9	30.2

**Table 4:** Porosity content of flexural specimens calculated from micro-CT scans.

Material	Porosity of Build Orientation (%)		
	*X*	*Y*	*Z*
PA11	1.24 ± 0.21	0.99 ± 0.19	0.86 ± 0.19
TPU	5.45 ± 0.30	3.53 ± 0.32	2.45 ± 0.68

**Table 5:** Tensile properties of the PA11 and TPU specimens.

Material	Orientation	UTS (MPa)	*ε*_ab_ (%)	Elastic Modulus (MPa)
PA11	*X*	46.0 ± 0.9	50.5 ± 6.4	932.4 ± 106.6
	*Y*	47.6 ± 0.5	49.9 ± 2.9	1116.5 ± 84.8
	*Z*	50.9 ± 0.4	32.1 ± 6.2	1319.8 ± 116.1
TPU	*X*	7.7 ± 0.6	222.3 ± 19.3	65.9 ± 5.8
	*Y*	8.8 ± 0.5	281.4 ± 35.8	75.6 ± 7.2
	*Z*	6.9 ± 0.2	133.8 ± 15.6	76.6 ± 3.5
SLS-printed PA11 [29]	*X*	48.6 ± 0.2	34.0 ± 2.0	1520.0 ± 70.0

**Table 6:** Flexural properties of the PA11 and TPU specimens.

Material	Build Orientation	Flexural Strength (MPa)	Flexural Modulus (MPa)
PA11	*X*	45.8 ± 1.7	961.2 ± 64.7
	*Y*	54.2 ± 3.7	1320.7 ± 142.9
	*Z*	58.0 ± 2.1	1444.2 ± 102.9
TPU	*X*	4.0 ± 0.1	66.1 ± 1.2
	*Y*	4.7 ± 0.1	77.8 ± 2.9
	*Z*	4.7 ± 0.2	76.3 ± 4.1
SLS-printed PA11 [10]	*X*	58.0 ± 2.0	1000.0 ± 100.0

**Table 7:** Tribological properties of the PA11 and TPU specimens.

Material	COF	Wear Volume (mm^3)^	Volume Loss (%)	Wear Rate (mm^3^/Nm)
PA11	0.13	0.082	0.002	8.68 × 10^−5^
TPU	0.55	11.74	0.30	0.012
SLS-printed PA12 [40]	0.18	-	-	6.61 × 10^−3^
